# Neuronal substrates for initiation, maintenance, and structural organization of sleep/wake states

**DOI:** 10.12688/f1000research.9677.1

**Published:** 2017-03-03

**Authors:** Ada Eban-Rothschild, Luis de Lecea

**Affiliations:** 1 Department of Psychiatry and Behavioral Sciences, Stanford University, Stanford, CA, 94305, USA

**Keywords:** sleep regulation, neuronal circuits, sleep transitions, NREM, REM, vigilance states

## Abstract

Animals continuously alternate between sleep and wake states throughout their life. The daily organization of sleep and wakefulness is orchestrated by circadian, homeostatic, and motivational processes. Over the last decades, much progress has been made toward determining the neuronal populations involved in sleep/wake regulation. Here, we will discuss how the application of advanced *in vivo *tools for cell type–specific manipulations now permits the functional interrogation of different features of sleep/wake state regulation: initiation, maintenance, and structural organization. We will specifically focus on recent studies examining the roles of wake-promoting neuronal populations.

## Introduction

Animals—including nematode worms ^1, 2^, bees ^3, 4^, flies ^5, 6^, fish ^7, 8^, rodents, humans, and even birds during migration ^9^—alter between wake and sleep states throughout their life. During wakefulness, animals engage in various adaptive and motivated behaviors related to foraging, courting, mating, and predator evading, among many others. Sleep is a state of quiescence with reduced responsiveness to external stimuli yet is restorative and recruits essential mechanisms for homeostatic balance ^10– 12^.

The daily organization of sleep and wake periods is orchestrated by circadian, homeostatic, and motivational processes ^13, 14^. The circadian clock (about 24 hours long) synchronizes sleep to an appropriate time of day; for example, night in diurnal animals and day in nocturnal animals. The homeostatic process is responsible for compensating sleep loss. In addition, environmental circumstances and internal needs, such as hunger ^9, 15– 18^, the presence of a predator ^19– 23^, or mating opportunities ^24^, can powerfully modulate sleep and wake states.

Sleep is ubiquitous in the animal kingdom, and the molecular pathways associated with sleep in the worm, fly, and mammals show much conservation, suggesting an ancient and common origin for sleep ^12, 25– 27^. For example, in both insects and mammals, histaminergic, noradrenergic, and dopaminergic neurotransmission promotes wakefulness whereas GABAergic and serotonergic neurotransmission promotes sleep ^28– 42^.

Sleep/wake disturbances are a major public health concern and affect 6% to 30% of the general adult population worldwide ^43^. Sleep disturbances have numerous deleterious effects, including impaired cognition, reduced immunity, and elevated risks of cancer and heart disease ^44, 45^. Perturbations of sleep/wake states are also associated with various neuropsychiatric disorders, such as major depression, substance abuse, and anxiety disorders ^45^. Increasing evidence suggests that several co-morbid pathologies found in neuropsychiatric disorders arise from a destabilization of sleep mechanisms ^44, 45^. Elucidating the neurobiological substrates of sleep and wakefulness could not only reveal how the brain orchestrates one of the most striking transitions in behavior and physiology, but could also provide a mechanistic framework for improved intervention with therapeutic purposes.

## Neuronal circuitry underlying the regulation of sleep/wake states

In mammals, birds, and reptiles, there are three general states of vigilance: wakefulness, non–rapid eye movement (NREM) sleep, and rapid eye movement (REM) sleep. The different states can be distinguished using electroencephalogram (EEG) and electromyogram recordings, which measure global cortical and muscular activity, respectively. The three vigilance states also differ in various physiological parameters, such as thermoregulation, brain metabolism, and breathing ^46^. How does the mammalian brain control sleep and wake states? von Economo ^47^, Ranson ^48^, and Moruzzi and Magoun ^49^ were among the first to examine a neuronal mechanism for sleep/wake regulation. Many subsequent studies have contributed to the identification of distinct neuronal populations across the brain that participate in sleep/wake regulation. It is currently understood that sleep/wake states are regulated by complex interactions between several neuronal populations, which show robust arousal state–dependent alterations in neuronal activity ^13^. Subcortical neuromodulatory neurons in the brainstem, midbrain, hypothalamus, and basal forebrain (BF) send widespread projections across the brain and interact with each other, the thalamus, and the cortex to drive behavioral, physiological, and electrocortical sleep/wake states ^13, 46, 50– 52^. In this review, we will focus mainly on wake-promoting populations. Key components of the arousal system are the following:

1) Monoaminergic neurons, including the noradrenergic locus coeruleus (LC) ^36, 53^, dopaminergic ventral tegmental area (VTA) ^31, 32^, dopaminergic and serotonergic dorsal raphe nucleus (DRN) ^41, 54– 56^, and histaminergic tuberomammillary nucleus (TMN) ^38, 57^ neurons.
2) Cholinergic neurons of the pedunculopontine and laterodorsal tegmental nuclei (PPT/LDT) ^58, 59^ and BF ^60, 61^.
3) Hypocretinergic (Hcrt, also known as orexinergic) neurons of the lateral hypothalamus (LH) ^62– 64^.

In each of these nuclei reside additional populations of GABAergic and glutamatergic neurons that have been shown to participate, or may participate, in sleep/wake regulation (for example, ^65^). A balance between the wake-promoting and the sleep-promoting neurons—such as the GABAergic neurons of the ventrolateral preoptic area and the median preoptic area ^66– 69^, the GABAergic neurons of the parafacial zone ^70, 71^, and melanin-concentrating hormone neurons of the LH ^72^—has been hypothesized as a theoretical model to understand sleep-to-wake transitions ^13^. According to this model, the mutually inhibitory interactions of wake-promoting and sleep-promoting neurons produce a state similar to a flip-flop switch in an electrical circuit ^13^.

From a functional dynamic perspective, one could identify neuronal circuits involved in the initiation, maintenance, and structural organization of the three vigilance states. With traditional strategies, such as brain lesions, pharmacological interventions, and animal knockout models, it was very difficult to address the causal role of specific populations in the regulation of the different components of vigilance states because they lacked both cellular specificity and temporal resolution. With the application of *in vivo* optogenetic ^73^ and chemogenetic ^74^ tools for cell type–specific neural manipulations and genetically encoded calcium indicators ^75^ for neural activity recordings, it is now possible to functionally interrogate the specific roles of, and interactions between, genetically defined neuronal populations across the brain in sleep/wake regulation.

## Initiation of vigilance states

Animals typically wake up rapidly—an adaptive response since they may need to flee or defend themselves when awakened. The transition between sleep and wakefulness has been hypothesized to involve fast glutamate transmission and wake-promoting neuromodulators ^13, 46, 76^. Optogenetic manipulations have demonstrated that increasing activity in noradrenergic LC ^77^, dopaminergic VTA ^31^, and cholinergic BF ^78– 81^ neurons during sleep can rapidly initiate wakefulness. Hcrt LH neurons have been hypothesized to regulate sleep-to-wake transitions based on homeostatic and environmental conditions ^82– 84^. Hcrt LH neurons are sensitive to diverse peripheral and central signals associated with nutritional state, such as low glucose (for example, ^85, 86^), and optogenetic stimulation of Hcrt LH neurons during NREM sleep increases the probability for a sleep-to-wake transition ^87^ only under low sleep pressure ^82^. Cholinergic neurons have long been suggested to play a critical role in cortical activation during both wakefulness ^88^ and REM sleep ^51^. Optogenetic stimulation of cholinergic BF neurons during NREM sleep can elicit a transition to either wakefulness or REM sleep ^79^, whereas optogenetic inhibition prolongs NREM sleep ^89^. These findings suggest that BF cholinergic neurons have an important role in NREM sleep termination, allowing the brain to transition to either wake or REM sleep ^89^.

Transitions from wakefulness to sleep are not instantaneous and can take a few seconds to minutes ^13^. With the initiation of NREM sleep, the EEG progressively changes from high-frequency, low-voltage waves characteristic of wakefulness, to higher-voltage, slower waves designating NREM sleep ^13, 46, 90^. Although the physiological and electrophysiological characteristics preceding and accompanying wake-to-NREM sleep transitions have been well studied ^90, 91^, relatively little is known about the neuronal underpinnings of naturalistic behaviors that precede sleep. Animals typically display species-specific behaviors prior to sleep ^92– 94^; they will search for a safe place, may build a nest, assume a specific body posture ^92– 94^, and engage in other behaviors, such as grooming and drinking ^95, 96^. A recent study demonstrated that mice drink prior to sleep in anticipation to the sleep period and not as a response to an immediate physiological need ^97^. Moreover, drinking prior to sleep is controlled by circadian output from the central clock in the suprachiasmatic nucleus to the organum vasculosum lamina terminalis neurons ^97^. We have recently identified a neuronal substrate for sleep-preparatory nest-building ^31^. We demonstrated that chemogenetic inhibition of VTA dopaminergic neurons promotes sleep, only in the presence of a nest. In the absence of a nest, the inhibition of VTA dopaminergic neurons first promoted nest-building and only later sleep. Taken together, these findings suggest that electrocortical sleep is coupled with preceding behavioral manifestations, yet the role of this preparatory phase in sleep structure and quality remains to be elucidated.

## Maintenance of vigilance states

During a normal sleep phase, animals continuously alternate between short periods of wakefulness, NREM sleep, and REM sleep. Typically, individuals enter NREM sleep from wakefulness and transition from NREM to either REM sleep or wakefulness. In humans, each cycle lasts around 90 minutes, whereas in rodents the cycles are shorter, lasting only several minutes. Once wakefulness, NREM sleep, or REM sleep is initiated, it is maintained for the duration necessary to fulfill its physiological purposes. How is the maintenance of vigilance states attained? One potential mechanism is continuous, tonic, or phasic activity in a certain neuronal population, such as histaminergic TMN neurons (for wakefulness). Activity in these neurons could directly support the maintenance of specific states, or inhibit the initiation of other vigilance states, by specific downstream projections. Another possibility, yet not mutually exclusive, is irregular phasic activity in a neuronal ensemble that initiates and supports a range of behavioral and physiological characteristics via various downstream targets. For example, Hcrt LH neurons are phasically active only during the transitions between sleep and wakefulness and during wakefulness when environmental conditions change ^63, 64^. This “kickstart” pattern of activity is likely widespread in arousal centers as it allows more adaptive responses to changing environments.

The different arousal systems vary in their capacity to promote wakefulness, and it has been hypothesized that the different neuronal populations have distinct roles in supporting arousal under specific environmental conditions ^51^. For example, histaminergic TMN neurons have an important role in maintaining arousal in novel environments ^38, 98^. Noradrenergic LC neurons promote attention and cognition during wakefulness ^99^ and have a pivotal role in supporting arousal in threatening circumstances ^100, 101^. Dopaminergic VTA neurons have a crucial role in wake maintenance in the face of various motivational processes, including mate- and food-seeking and predator evading ^31^. Serotonergic DRN neurons have been suggested to support quiet wakefulness, possibly preceding sleep initiation ^51, 102, 103^. A distinct role for each wake-related neuronal population in promoting distinct forms of arousal under specific environmental conditions could clarify the relatively surprising redundancy in wake-promoting circuits ^13, 51^.

It is important to note that specific sleep/wake regulatory populations could have a more complex role than supporting one vigilance state. Histaminergic TMN neurons that have long been implicated in wake maintenance via histamine neurotransmission ^13^ also release GABA, which rather seems to promote sleep ^104^, at least via some projections. The co-transmission of histamine and GABA could serve as a break to the wake-promoting effects of histamine ^104^. It would be of interest for future studies to further determine the importance of co-transmission in additional sleep/wake neuronal populations and the precise role the neuromodulatory substrates by themselves play in sleep/wake regulation.

## Structural organization of vigilance states

Another important feature of sleep/wake regulatory circuits is maintaining the boundaries between vigilance states. A failure to maintain these boundaries could have severe consequences for survival if, for instance, a predator defense behavior were interrupted by an unexpected transition to sleep. In addition, the restorative and memory consolidation functions of sleep are dependent upon proper consolidation of sleep, as demonstrated by the deleterious effects of sleep fragmentation ^43, 105^.

The daily organization of sleep and wake periods is orchestrated by circadian, homeostatic, and motivational processes. The circadian clock (about 24 hours long) synchronizes sleep to an appropriate time of day (that is, night in diurnal animals and day in nocturnal animals). The homeostatic process is responsible for compensating sleep loss following sleep debt. In addition, environmental circumstances and internal needs, here referred to as “motivational processes”, can powerfully affect sleep/wake states.

How do regulatory circuits maintain the boundaries between the vigilance states? The hypocretin system is hypothesized to orchestrate the structural organization of sleep/wake states ^84, 106^. The hypocretins are two neuropeptides, Hcrt-1 and Hcrt-2, produced from the pre-pro-hypocretin precursor, which are expressed solely in a glutamatergic neuronal population in the LH. Hcrt neurons project to diverse areas of the central nervous system, including to major sleep/wake nuclei, such as the LC, TMN, DRN, PPT, LDT, and VTA ^62, 107^, that express the Hcrt receptors, Hcrt-R1 and Hcrt-R2 ^108^. *In vitro* electrophysiology and histological studies demonstrate that Hcrt neurons are activated by neurotransmitters that promote arousal, including corticotropin-releasing factor ^109^ and thyrotropin-releasing hormone ^110^, and inhibited by sleep-promoting substances, including GABA ^111^ and adenosine ^112^.

Hcrt LH neurons are essential for the stability of arousal and malfunction of the Hcrt network fragments sleep and wake states. The loss of Hcrt neurons, or its receptors, in rodents ^113– 115^, canines ^116^, and humans ^117– 119^ is associated with narcolepsy with cataplexy, a neurological disorder characterized by an inability to control the boundaries between sleep/wake states. In narcoleptics, periods of wakefulness are interrupted by unexpected sleep episodes, and REM-like episodes coexist with conscious wakefulness ^120^. Similarly, Hcrt knockout or Hcrt-R2-deficient mice show increased arousal state–transitions but do not vary in the total daily duration of sleep and wake states from control animals. Lastly, optogenetic stimulation of Hcrt LH neurons during sleep, in rodents, increases the probability for a sleep-to-wake transition ^87^. Together, these findings support the premise that under physiological conditions Hcrt LH neurons are important in maintaining the boundaries between sleep/wake states.

It is also important to note that the three vigilant states are not always mutually exclusive, and different dissociated states exist in humans as well as other animals. Slow-wave sleep can occur locally in cortical areas ^121– 123^ as well as in individual neurons while animals are behaviorally awake ^124^. In addition, unihemispheric slow-wave sleep (USWS) has been documented in a number of aquatic mammals ^125^ and birds ^126^. During USWS, the eye contralateral to the awake hemisphere is open and could monitor the environment. This plasticity could permit birds to defend themselves from predators or continuously fly during long migration periods and aquatic mammals to breathe or take care of their young during critical periods ^126^.

## Conclusions and perspectives

During the last decade, major advances have been made in characterizing the neuronal populations participating in sleep/wake regulation. However, it is still unclear how the brain integrates information from diverse populations to control overt arousal. Are the different arousal populations promoting wakefulness in different ecological contexts? How does the brain prioritize arousal based on environmental circumstances and homeostatic needs? In addition, future studies should further examine the role of distinct subpopulations of GABAergic, glutamatergic, and peptidergic neurons in sleep/wake regulatory nuclei.
